# Systemic S100A8/A9 in patients with moderate to severe acute ischemic stroke: Exploratory analysis of inflammation and functional outcome

**DOI:** 10.1016/j.bbih.2025.101041

**Published:** 2025-06-18

**Authors:** Christoph Vollmuth, Felipe A. Montellano, Cornelia Fiessler, Fabian Essig, Christian Hametner, Alexander M. Kollikowski, Vivian Vogt, Mirko Pham, Peter U. Heuschmann, Karl Georg Haeusler, Guido Stoll, Hermann Neugebauer, Michael K. Schuhmann

**Affiliations:** a University Hospital Würzburg (UKW), Department of Neurology, Würzburg, Germany; b University of Würzburg, Institute for Clinical Epidemiology and Biometry, Würzburg, Germany; c University Hospital Würzburg (UKW), Department of Neuroradiology, Würzburg, Germany; d Institute for Medical Data Science, University Hospital Würzburg, Germany; e Clinical Trial Centre, University Hospital Würzburg, Germany; f University Hospital Ulm, Department of Neurology, Ulm, Germany; g Institute of Experimental Biomedicine I, University Hospital Würzburg, Germany

**Keywords:** Blood-based biomarker, S100A8/A9, Calprotectin, Inflammation, Prognosis, Danger-associated molecular patterns

## Abstract

**Introduction:**

S100A8/A9, a danger-associated molecular pattern (DAMP), is released from leukocytes, mainly neutrophils, and augments inflammation and tissue damage. The role of systemic plasma levels of S100A8/A9 in stroke-related inflammation and its association with clinical outcome lacks sufficient data.

**Methods:**

Prospective, monocentric, observational study including patients with moderate to severe acute ischemic anterior circulation stroke [National Institutes of Health Stroke Scale (NIHSS) score ≥6 points and/or mechanical recanalization)]. We assessed functional outcome by telephone interview 3 months (±14 days) after stroke using the 7-point modified Rankin Scale (mRS). Poor outcome was defined as mRS ≥3. Systemic plasma levels of S100A8/A9 were determined by ELISA <48 h after onset of symptoms, alongside a differential blood count. Univariable and multivariable logistic regression were performed to investigate the association between systemic plasma levels of S100A8/A9 and functional outcome.

**Results:**

Between June 2020 and September 2022, a total of 272 patients were enrolled [52 % female, median age 79 years (IQR: 66–84), median NIHSS score on admission 13 (IQR: 8–17), median ASPECTS 8 (IQR: 6–9)]. Of these, 220 patients (81 %) underwent mechanical recanalization, and 118 (43 %) received systemic thrombolytic therapy. There was a significant correlation between systemic plasma levels of S100A8/A9 and neutrophil counts at baseline [p < 0.0001; r = 0.33 (95 % confidence interval: 0.22; 0.44)]. At 3 months, 192 of 272 (71 %) patients had poor functional outcome, who had significantly higher systemic plasma levels of S100A8/A9 at baseline [median: 525 ng/ml (IQR: 342–897)] than those with good functional outcome [397 ng/ml (IQR: 232–580); p = 0.001]. Furthermore, systemic plasma levels of S100A8/A9 at baseline were associated with poor outcome [unadjusted odds ratio (OR): 2.01 (95 %CI: 1.04–3.96)], however this association was attenuated and no longer significant when adjusting for age, sex, NIHSS Score on admission, ASPECT Score on admission and recanalization therapy (yes/no) [adjusted OR: 1.92 (95 %CI: 0.86–4.34)].

**Conclusions:**

Systemic plasma levels of S100A8/A9 were associated with poor outcome in patients with moderate to severe ischemic stroke. The observed correlation with neutrophil counts at baseline might underscore an important pathophysiological link between patients’ prognosis and stroke-related inflammation.

**Study registration:**

DRKS00022064.

## Non-standard abbreviations and acronyms

ASPECTS: Alberta Stroke Program CT Score
eTICI: expanded Treatment in Cerebral Ischemia
mRS: modified Rankin Scale
NIHSS: National Institutes of Health Stroke Scale
TOAST: Trial of ORG 10172 in Acute Stroke Treatment

## 1 Introduction

Despite successful revascularization through systemic thrombolysis and/or mechanical thrombectomy, half of the patients with severe acute ischemic stroke (AIS) still experience unfavorable functional outcomes (Wollenweber et al., 2019). Neuroinflammation has long been recognized as a significant aspect of stroke, but its potential relevance has only recently become evident (Stoll and Nieswandt, 2019). The molecular mechanisms governing the initial inflammatory responses in acute stroke and their implications for stroke outcome remain largely elusive.

S100A8/A9 (calprotectin) has emerged as a significant prognostic biomarker and mediator in various inflammatory conditions, particularly in cardiovascular and cerebrovascular diseases. This heterodimer, composed of the S100 family proteins S100A8 and S100A9, is predominantly expressed in myeloid cells, particularly neutrophils (Joshi et al., 2022; Wang et al., 2023). In middle-aged individuals, plasma S100A8/A9 levels correlate with blood neutrophil counts and traditional cardiovascular risk factors, indicating its potential as a biomarker and mediator of neutrophil involvement in cardiovascular disease (Cotoi et al., 2014). Elevated S100A8/A9 levels have been observed in patients with myocardial infarction (Frangogiannis, 2019; Schiopu and Cotoi, 2013) and deep vein thrombosis (Wang et al., 2017).

Within the first 24 h following a stroke, considered the hyperacute phase of ischemic stroke and often referred to as the “golden window” for treatment, recent studies have highlighted a significant local intravascular inflammatory response within collateral vessels nourishing the ischemic penumbra under large vessel occlusion. The response is characterized by neutrophil recruitment and platelet activation (Stoll et al., 2022). This neutrophil recruitment is associated with the release of danger-associated molecular patterns (DAMPs) such as S100A8/A9 (Schuhmann et al., 2021a). Elevated levels of S100A8/A9 in venous blood samples have been correlated with functional outcome and increased 3-months mortality in patients with mild to moderate AIS suggesting its potential as a prognostic biomarker in human ischemic stroke (Marta-Enguita et al., 2021; Guo et al., 2020). Marta-Enguita et al. analyzed 413 patients with mild to moderate stroke, defined by a National Institutes of Health Stroke Scale (NIHSS) score of 6 (IQR 3–15), with blood samples obtained within 24 h of hospital admission. Similarly, Guo D et al. examined 4785 patients, divided into two cohorts of mild to moderate stroke. The first cohort (1322 subjects) had a baseline NIHSS score of 5 (IQR 3–8), and the second cohort (3463 subjects) had a baseline NIHSS score of 4 (IQR 2–8); both groups provided fasting blood samples collected within 24 h of admission. Despite these findings, it remains unclear whether systemic plasma levels of S100A8/A9 independently predict poor outcomes in patients with moderate to severe AIS.

The aim of this study was to investigate the association between systemic plasma levels of S100A8/A9 and neutrophil counts during the acute phase of stroke in patients with moderate to severe AIS. Additionally, we aimed to explore the relationship between plasma S100A8/A9 levels and functional outcome 3-months after-stroke, assessing its role in inflammation-associated morbidity in AIS.

## 2 Methods

In this prospective, investigator-initiated single-center observational cohort study, we recruited patients presenting with moderate to severe AIS in the anterior circulation, as indicated by a National Institutes of Health Stroke Scale (NIHSS) score of ≥6 points upon admission and/or attempt of mechanical recanalization due to occlusion of a large intracranial vessel (regardless of their NIHSS score). Ischemic stroke was defined according to WHO criteria. Blood samples were required to be taken within 48 h of symptom onset. Exclusion criteria included age under 18 years and insufficient proficiency in German. Additionally, we excluded individuals who had previously participated in trials that could potentially affect platelet function or stroke outcomes within the past 3 months.

### 2.1 Ethical Approval

This study is in accordance with the Declaration of Helsinki and its later amendments. Written informed consent was obtained from all participants or legal representatives. The study was assessed and approved by the local Ethics Committee of the University of Würzburg (reference n. 05/20-am). The study was registered under DRKS00022064.

### 2.2 Central clinical database

Data were assessed in a centralized database, ensuring comprehensive documentation of all demographic and clinical variables. National Institutes of Health Stroke Scale (NIHSS) scores were meticulously evaluated by seasoned neurologists upon admission, at 24, 48, and 72 h, and at the time of hospital discharge. Early infarct signs in non-contrast CT scans on admission were evaluated independently by experienced neuroradiologists using the Alberta Stroke Program Early CT Score (ASPECTS). Additionally, expanded Treatment in Cerebral Ischemia (eTICI) scores were independently assessed by experienced neuroradiologists. The etiology of ischemic stroke was classified based on the Trial of ORG 10172 in Acute Stroke Treatment (TOAST) criteria using information available at the time of discharge. Neurological disability was assessed using the modified Rankin Scale (mRS) both before admission and at hospital discharge. Functional outcome was determined through structured telephone interviews conducted by a blinded assessor at 3 months (±14 days) post stroke. A favorable functional outcome was defined as a mRS score of 0–2, while a poor outcome was categorized as a mRS score of 3–6. Additionally, cardiovascular risk factors and comorbidities were elucidated through patient interviews and thorough chart reviews.

### 2.3 Blood sampling and analysis

Blood was drawn in the morning after enrollment from the antecubital vein. After application of a tourniquet, venous puncture was performed with a 21G Multifly adapter (Sarstedt, Germany). The tourniquet was then released and blood was drawn by aspiration technique in a Citrat 4.3 ml S-Monovette (Sarstedt, Germany) and then spun down at 2500×*g* for 10 min. Samples were aliquoted with 360 μl per tube into 1.0 storage tubes. Tubes were then registered to the rack and a patient pseudonym in a custom programmed Access Database. Samples were then stored at −80 °C. All plasma samples analyzed were not subjected to repeated freeze-thaw cycles. The plasma levels of S100A8/A9 were measured using the Thermo Scientific Human Calprotectin L1/S100-A8/A9 Complex Enzyme-Linked Immunosorbent Assay Kit (EH62RB), which has a detection limit of 35 pg/ml.

### 2.4 Statistical analysis

Statistical analysis was performed using R 4.2.2 (R Foundation, Vienna, Austria) and GraphPad 10 (GraphPad Software, La Jolla, USA). Patients with elevated systemic plasma levels of S100A8/A9 were identified as outliers and excluded using the ROUT test with a statistical threshold of p < 0.01. Categorical variables were reported as numbers of patients with percentage of the total cohort (%) and continuous variables with normal distributions were reported as mean with standard deviation (SD), while non-normally distributed variables were presented as median with interquartile range (IQR). For two-group comparisons of categorical and continuous variables the χ2 test, Student's t-test, and Mann–Whitney *U* test was used, according to the distribution of variables. The normality of distributions was initially assessed using graphical methods, including histograms and Q-Q plots (quantile-quantile plots), and further evaluated with statistical tests, such as the D'Agostino-Pearson test, in cases of uncertainty (Ghasemi and Zahediasl, 2012). Correlations between biomarker levels were calculated with the Spearman's coefficient. Multicollinearity was assessed by examination of the correlation matrix and variables with associations ≥0.8 were omitted. For logistic regression, biomarker levels were logarithmically transformed due to skewed distribution. We report odds ratios (OR) with corresponding 95 % confidence intervals (CI) to changes per log-unit. Associations of blood-based biomarkers with poor outcome was assessed using logistic regression. Adjustment was conducted for variables that have been identified as the most important covariates [(age, sex, NIHSS Score on admission, ASPECT Score on admission and recanalization therapy (yes/no)] [(age, sex, NIHSS Score 24 h after hospital admission, ASPECT Score and recanalization therapy (yes/no)] (Montellano et al., 2021; Brugnara et al., 2020). We assessed discrimination using the area under the receiver operator curve (AUROC). Additionally, we calculated the cut-off value for systemic plasma levels of S100A8/A9 using the Youden index. P values < 0.05 were considered to indicate statistical significance. All tests were performed two-tailed.

## 3 Results

### 3.1 Baseline characteristics

**Fig. 1:**
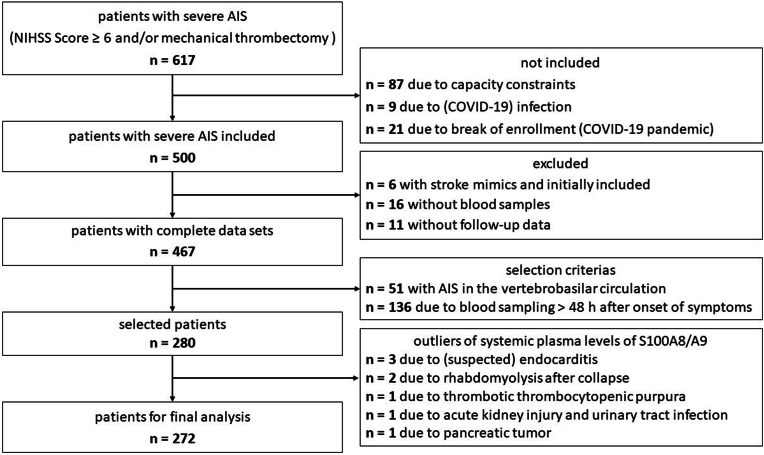
Final study population selection process.

Between June 2020 and September 2022, 617 patients with moderate to severe AIS were admitted to our tertiary care center (University Hospital Würzburg, Würzburg, Germany). However, due to a temporary halt in enrollment caused by the COVID-19 pandemic between December 2020 and January 2021, 21 stroke patients treated during that period were not included in the study. Additionally, 87 patients were excluded due to capacity constraints, and 9 patients were excluded due to COVID-19 infection. Of the 500 patients initially enrolled, 6 were later excluded due to stroke mimics. Blood samples could not be obtained from 16 patients, and 11 patients did not participate in follow-up. Furthermore, 51 patients who experienced AIS in the vertebrobasilar circulation were excluded due to selection criteria, along with 136 patients whose blood sampling occurred more than 48 h after symptom onset. Eight patients were excluded as outliers with high S100A8/A9 levels (3833–12509 ng/ml). Among these, two patients were diagnosed with endocarditis, and one was suspected of having it. Additionally, one patient presented with pancreatic head carcinoma, two with rhabdomyolysis after collapse, one with acute renal failure due to a urinary tract infection and one with thrombotic thrombocytopenic purpura. Consequently, a total of 272 patients were included in the analysis (Fig. 1).

**Fig. 2:**
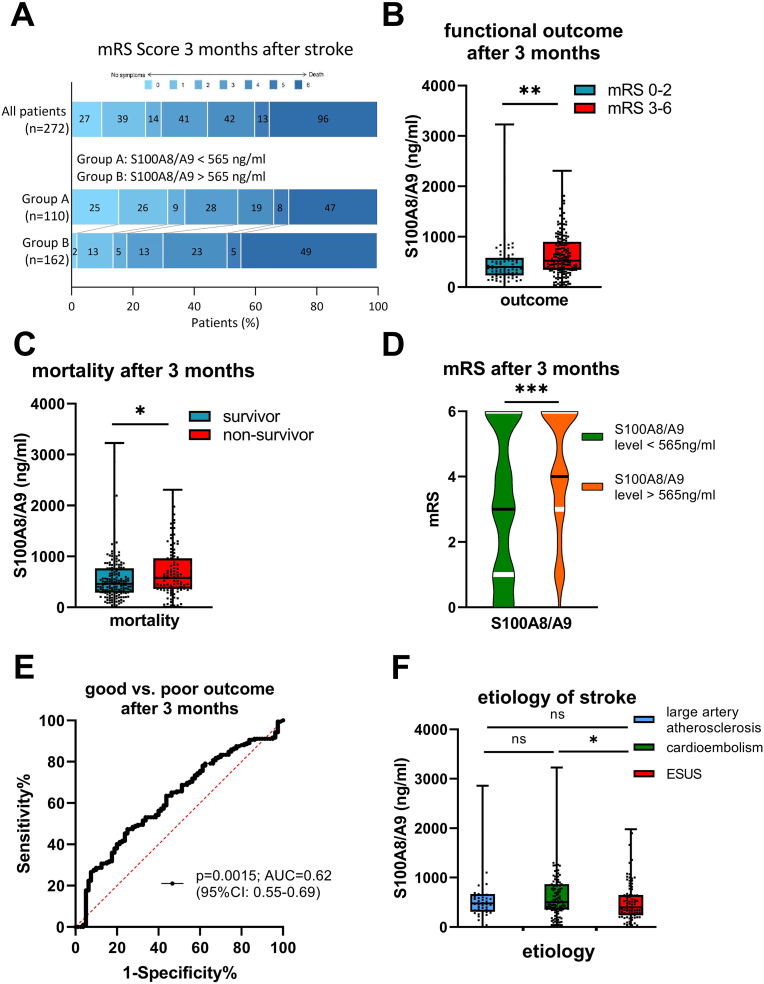
Prognostic value of systemic plasma levels of S100A8/A9 in terms of stroke severity and mortality to predict 3-months outcome: **A)** Functional outcome after three months of all patients and after stratifying patients by cut-off value using the Youden index (565 ng/ml). **B)** S100A8/A9 according to 3-month functional outcome (good vs. poor outcome). **C)** Survival at 3-months follow-up. **D)** mRS at 3-months follow-up according to cut-off value of systemic plasma levels of S100A8/A9 using the Youden index (cut-off: 565 ng/ml) (the black line represents the median and the white lines indicate the interquartile range). **E)** ROC analysis for 3-months functional outcome (mRS 0–2 vs. 3–6). **F)** S100A8/A9 according etiology of stroke [large artery atherosclerosis, cardioembolism, embolic stroke of undetermined source (ESUS)]. ∗p-value<0.05; ∗∗p-value<0.01; ∗∗∗p-value<0.001.

Overall, 272 patients [52 % female, median age 79 years (IQR: 66; 84), median NIHSS score on admission 13 (IQR: 8; 17), median ASPECTS on admission 8 (IQR: 6; 9)] with complete biomarker and follow-up information were included in the study. A total of 220 (81 %) patients underwent mechanical thrombectomy and 118 (43 %) systemic thrombolytic therapy, including 90 (33 %) patients undergoing both treatments (Table 1). Out of 272 patients, 80 (29 %) had a good functional outcome, while 192 (71 %) had a poor functional outcome 3-months after stroke (Fig. 2A). Among those with a poor outcome, 96 patients (50 %) died, with 74 (77 %) dying in the hospital and 22 (23 %) dying after discharge. With the exception of diabetes mellitus, no significant differences were observed between the groups (good vs. poor outcome at 3 months) in terms of vascular risk factors (hypertension, heart failure, atrial fibrillation) or stroke etiology.

**Table 1:** Clinical, radiological and biochemical data of the study population.

Table 1			total (n = 272)	mRS 0–2 at 3 months (n = 80)	mRS 3–6 at 3 months (n = 192)	p-value^c^
baseline data						
	age, years, median [IQR]		79 [66–84]	72 [62–81]	80 [69–85]	**<0.0001**
	sex, female, n (%)		142 (52.2)	26 (32.5)	116 (60.4)	**<0.0001**
	pre stroke mRS 0–2, n (%)		223 (82.0)	80 (100)	143 (74.5)	**<0.0001**
	creatinin, mg/dl, median [IQR]		0.96 [0.8–1.2]	0.93 [0.77–1.11]	0.96 [0.79–1.28]	0.12
	time from stroke onset to follow up, days, median [IQR]		90 [84–97]	87 [82–95]	92 [85–98]	0.0092
vascular risk factors, n (%)						
	hypertension		175 (64.3)	45 (56.3)	130 (67.7)	0.10
	diabetes mellitus		54 (19.9)	9 (11.3)	45 (23.4)	**0.029**
	heart failure		35 (12.9)	6 (7.5)	29 (15.1)	0.11
	atrial fibrillation		122 (44.9)	34 (42.5)	88 (45.8)	0.69

stroke severity^a^, median [IQR]						
	NIHSS Score on admission		13 [8–17]	10 [7–16]	14 [10–18]	**<0.0001**
	NIHSS Score at 24h		12 [4–20]	3 [1–6]	16 [9–17]	**<0.0001**
	NIHSS Score at 48h		9 [3–18]	2 [1–4]	14 [7–22]	**<0.0001**
	NIHSS Score at 72h		7 [2–16]	2 [0–3]	13 [6–20]	**<0.0001**
	NIHSS Score at discharge^a^		4 [1–10]	1 [0–2]	8 [3–13]	**<0.0001**
etiology, n (%)						
	large artery atherosclerosis		48 (17.6)	17 (21.3)	31 (16.1)	0.38
	cardioembolism		123 (45.2)	35 (43.8)	88 (45.8)	0.79
	cryptogenic		87 (32.0)	25 (31.3)	62 (32.3)	0.89
	other causes		14 (5.1)	3 (3.8)	11 (5.7)	0.57

neuoradiologic data						
blood vessel occluded, n (%)						
		Carotis-T	28 (10.3)	2 (2.5)	26 (13.5)	**0.007**
		M1	164 (60.3)	43 (53.8)	121 (63.0)	0.17
		M2	58 (21.3)	24 (30.0)	34 (17.7)	**0.034**
ASPECT Score, median [IQR]						
		ASPECT Score on admission	8 [6–9]	9 [8–9]	7 [6–8]	**<0.0001**

acute treatment, n (%)						
	thrombolysis		118 (43.4)	42 (52.5)	76 (39.6)	0.06
	time from onset to thrombolysis, hours, Median [IQR]		1.7 [1.2–2.4]	1.5 [1.2–2.3]	1.8 [1.3–2.6]	0.42
	mechanical thrombectomy		220 (80.9)	59 (73.6)	161 (83.9)	0.06
	time from onset to mechanical thrombectomy, hours^b^, Median [IQR]		4.2 [2.7–8.7]	3.6 [2.2–6.2]	4.5 [3.1–11.7]	**0.029**
	TICI ≥ 2b		193 (87.7)	58 (98.3)	135 (83.9)	**0.004**

prior medication, n (%)						
	inhibitor of platelet aggregation		120 (44.1)	40 (50.0)	80 (41.7)	0.23
	acetylsalicylic acid		113 (41.5)	39 (48.8)	74 (38.5)	0.14
	clopidogrel		15 (5.5)	3 (3.8)	12 (6.3)	0.56
blood sample and biomarker data						
	time from onset to blood sample, hours, Median [IQR]		27 [20–37]	25 [19–35]	28 [20–38]	0.14
differential blood count, median [IQR]						
	thrombocytes × 10^3^/μl		191 [149–233]	186 [147–235]	194 [149–233]	0.71
	leukocytes × 10^3^/μl		9.3 [7.6–11.5]	9.1 [7.4–10.4]	9.5 [7.8–12.1]	**0.013**
	monocytes × 10^3^/μl		0.7 [0.6–0.9]	0.64 [0.55–0.87]	0.75 [0.58–0.98]	**0.034**
granulocytes						
		eosinophiles × 10^3^/μl	0.1 [0.0–0.1]	0.1 [0.0–0.1]	0.0 [0.0–0.1]	**0.013**
		neutrophiles × 10^3^/μl	7.0 [5.5–9.2]	6.4 [5.2–8.3]	7.3 [5.8–9.5]	**0.006**
	lymphocytes × 10^3^/μl		1.2 [0.8–1.6]	1.3 [0.99–1.62]	1.1 [0.72–1.5]	**0.013**
	neutrophil-to-lymphocyte ratio		6.3 [3.8–10.5]	5.0 [3.3–7.1]	6.7 [4.2–11.3]	**0.0005**

biomarker data, median [IQR]						
	S100A8/A9, ng/ml		487 [312–829]	397 [232–580]	525 [342–897]	**0.001**
		with antiplatelet agents	512 [287–786]	359 [199–617]	557 [341–933]	**0.013**
		without antiplatelet agents	465 [337–835]	400 [274–556]	519 [344–882]	0.064

a Deceased patients excluded.

b Only patients with mechanical thrombectomy.

c Patients with good functional outcome vs. poor outcome.

### 3.2 Univariable analysis of systemic plasma levels of S100A8/A9 at baseline and functional outcome

Patients with poor functional outcome had significantly higher systemic plasma levels of S100A8/A9 [median: 525 ng/ml (IQR: 342–897)] than those with good functional outcome [median: 397 ng/ml (IQR: 232–580), p = 0.001] (Fig. 2B). Further, we explored the association between systemic plasma levels of S100A8/A9 and mortality at 3-month follow-up. Comparing the two groups, non-survivors [median: 577 ng/ml (IQR: 363–962)] showed increased systemic plasma levels of S100A8/A9 compared to survivors [median: 487 ng/ml (IQR: 287–766), p = 0.028] (Fig. 2C). Furthermore, we determined the cut-off value for systemic plasma levels of S100A8/A9 predicting poor functional outcome using the Youden index (cut-off: 565 ng/ml). When stratifying patients by this cut-off value (565 ng/ml), those with higher baseline biomarker levels exhibited significantly higher mRS scores at the 3-month follow-up [4 (IQR: 3–6) vs. 3 (IQR: 1–6); p < 0.0001] (Fig. 2A+2D and Table S1).

### 3.3 Univariable analysis of systemic plasma levels of S100A8/A9 at baseline and baseline parameters

We distinguished patients according to the cut-off value of systemic plasma level of S100A8/A9 (cut-off: 565 ng/ml) at baseline. Between patients with high and low systemic plasma levels of S100A8/A9 there were no significant differences in NIHSS scores on admission [13 (IQR: 8–17) vs. 13 (IQR: 9–17); p = 0.091], while there was a significant difference between the groups at day 1 [14 (IQR: 7–23) vs. 8 (IQR: 3–18); p = 0.0003], day 2 [12 (IQR: 5–21) vs. 6 (IQR: 2–16); p = 0.0004] and day 3 [11 (IQR: 4–17) vs. 4 (IQR: 1–14); p = 0.0003], and at discharge [5 (IQR: 2–11) vs. 2 (IQR: 1–7), p = 0.0010] (Table S1). Furthermore, no statistically significant difference was observed between patients with high and low systemic plasma levels of S100A8/A9 (cut-off: 565 ng/ml) in terms of the ASPECT score on admission [7 (IQR: 6–8) vs. 8 (IQR: 7–9), p = 0.068] (Table S1).

### 3.4 Discrimination and correlations of systemic plasma levels of S100A8/A9 with functional outcome, baseline characteristics and differential blood count

**Fig. 3:**
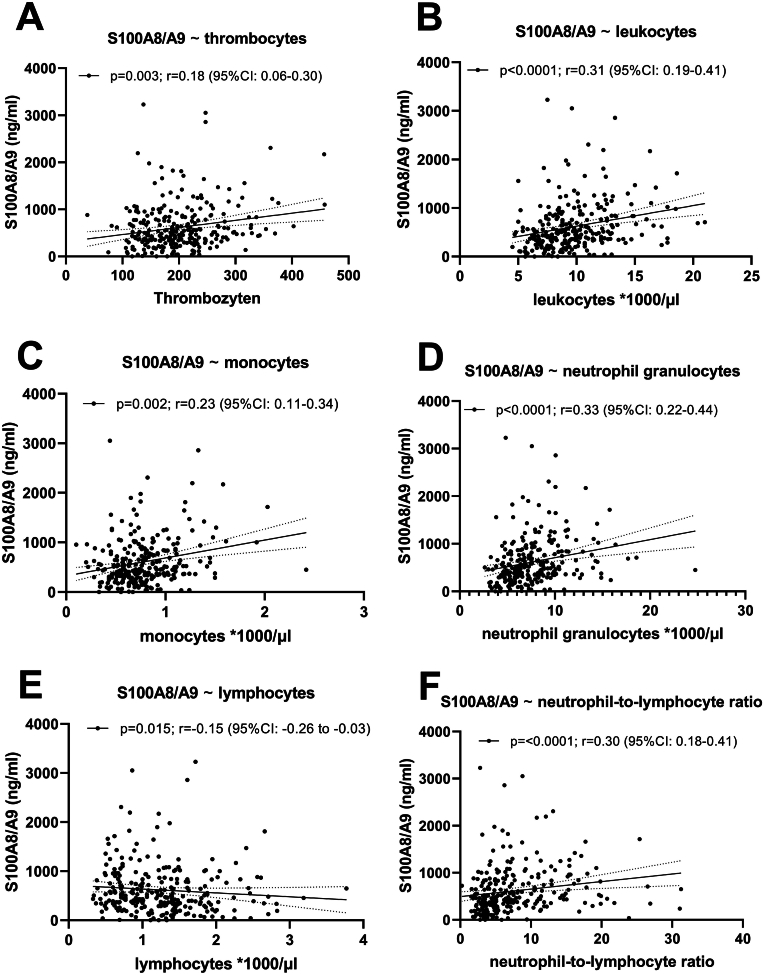
Spearman's correlations among systemic plasma levels of S100A8/A9 and differential blood count: **A)** thrombocytes **B)** leukocytes **C)** monocytes **D)** neutrophil granulocytes **E)** lymphocytes **F)** neutrophil-to-lymphocyte ratio.

The ROC-curve predicting poor functional outcome (mRS 0–2 vs. 3–6) showed an area under the curve of 0.62 (p = 0.0015; 95 %CI: 0.55–0.69) (Fig. 2E). In addition, we found significant correlations between systemic plasma levels of S100A8/A9 and thrombocytes [p = 0.003; r = 0.18 (95 %CI: 0.06–0.30)], leukocytes [p < 0.0001; r = 0.31 (95 %CI: 0.19–0.41)], monocytes [p = 0.0002; r = 0.23 (95 %CI: 0.11–0.34)], neutrophil granulocytes [p < 0.0001; r = 0.33 (95 %CI: 0.22–0.44)], lymphocytes [p = 0.015; r = −0.154 (95 %CI: 0.26 to −0.03)] as well as the neutrophil-to-lymphocyte-ratio [p < 0.0001, r = 0.30 (95 %CI: 0.18–0.41)] (Fig. 3A–F, Table 2).

**Table 2:** Correlation of systemic plasma levels of S100A8/fA9 with differential blood count.

variable		systemic plasma levels of S100A8/A9 at baseline	
		p-value	r [95 % CI]
differential blood count			
	thrombocytes	**0.003**	0.18 [0.06–0.30]
	leukocytes	**<0.0001**	0.31 [0.19–0.41]
	monocytes	**0.0002**	0.23 [0.11–0.34]
	neutrophil granulocytes	**<0.0001**	0.33 [0.22–0.44]
	lymphocytes	**0.015**	−0.15 [-0.26 to −0.03]
	neutrophil-to-lymphocyte ratio	**<0.0001**	0.30 [0.18–0.41]

### 3.5 Univariable and multivariable regression of systemic plasma levels of S100A8/A9 with baseline characteristics and differential blood count

Results of the unadjusted and adjusted regression analysis are shown in Table 3. In the unadjusted regression analysis, systemic plasma levels of S100A8/A9 were associated with poor functional outcome [odds ratio (OR): 2.01 (95 % CI: 1.04–3.96)]. However, after adjusting for age, sex, NIHSS Score on admission, ASPECT Score on admission, and recanalization therapy (yes/no), this association was attenuated and no longer significant [OR: 1.92 (95 %CI: 0.86–4.34)]. Similarly, after adjustment for NIHSS Score 24 h after admission instead of NIHSS Score on admission, systemic plasma levels of S100A8/A9 showed no significant association with poor outcome [OR: 1.40 (95 % CI: 0.50–3.87)]. Furthermore, we analyzed the association between neutrophil count, the neutrophil-to-lymphocyte ratio, and functional outcome. The results of both the univariable and multivariable analyses are presented in the supplementary materials.

**Table 3:** Univariable and multivariable analysis for association of systemic plasma levels of S100A8/A9 and functional outcome 3-months after stroke.

variable	Odds ratio^a^	Odds ratio^b^	Odds ratio^c^
	[95 % CI]	[95 % CI]	[95 % CI]
Age	1.04 [1.02; 1.06]	1.05 [1.02; 1.08]	1.05 [1.01; 1.08]
Sex	3.17 [1.85; 5.56]	2.09 [1.11; 3.97]	3.04 [1.46; 6.49]
NIHSS Score on admission	1.13 [1.08; 1.20]	1.09 [1.03; 1.16]	
NIHSS Score after 24h	1.25 [1.18; 1.34]		1.23 [1.15; 1.32]
ASPECT Score	0.20 [0.11; 0.36]	0.20 [0.09; 0.39]	0.36 [0.16; 0.79]
Recanalization therapy (yes/no)	0.61 [0.20; 1.57]	0.57 [0.16; 1.88]	0.64 [0.15; 2.47]
Log(S100A8/A9)	2.01 [1.04; 3.96]	1.92 [0.86; 4.34]	1.40 [0.50; 3.87]
Odds ratios are reported for logarithmic increases of base 10.			

a Univariable analysis.

b Adjusted for age, sex, NIHSS on admission, ASPECTS and recanalization therapy (yes/no).

c Adjusted for age, sex, NIHSS 24 h after admission, ASPECTS and recanalization therapy (yes/no).

### 3.6 Association of systemic plasma levels of S100A8/A9 with etiology of stroke

By categorizing stroke patients based on stroke etiology [large artery atherosclerosis, cardioembolism, and embolic stroke of undetermined source (ESUS)], we found that patients with cardioembolic stroke had significantly higher systemic plasma levels of S100A8/A9 compared to those with ESUS [median: 506 ng/ml (IQR: 353–868) vs. median: 398 ng/ml (IQR: 239–647), p = 0.02] (Fig. 2F). However, no significant difference in S100A8/A9 levels was observed between patients with large artery atherosclerosis [median: 477 ng/ml (IQR: 307–670)] and those with either cardioembolism (p = 0.39) or ESUS (p = 0.26) (Hart et al., 2014).

## 4 Discussion

This study explores the role of S100A8/A9, a neutrophil derived plasma biomarker, in stroke-related inflammation and its association with clinical outcomes. We show that elevated levels of systemic S100A8/A9 are significantly associated with poor functional outcome in patients with moderate to severe ischemic stroke, and that these levels correlate with neutrophil counts, suggesting a potential mechanistic link between neutrophil-driven inflammation and stroke prognosis. Inflammatory processes are increasingly recognized as critical contributors to lesion progression in ischemic stroke. In this context, damage-associated molecular patterns (DAMPs), such as S100A8/A9 and HMGB1, have emerged as key mediators of post-ischemic inflammation, offering promising avenues for both diagnostic biomarkers and therapeutic intervention. Previous studies have shown an association between systemic plasma levels of S100A8/A9 at baseline and functional outcomes 3 months after stroke with mild to moderate severity (Marta-Enguita et al., 2021). This finding aligns with our study cohort, which in contrast includes patients with moderate to severe ischemic stroke, characterized by a median NIHSS score of 13 on admission.

Moreover, the observed association between systemic plasma levels of S100A8/A9 and mortality within three months post stroke underscores the potential as an indicator of poor or limited prognosis. In addition, systemic plasma levels of S100A8/A9 have been shown to be associated with short-term functional outcomes (NIHSS score 24 h after hospital admission) (Hu et al., 2022), which was likewise demonstrated in our study. However, the ROC curve of the study presented here showed a lower area under the curve compared to other biomarkers like serum neurofilament light chain and glial fibrillary acidic protein (Vollmuth et al., 2024).

While univariable regression analysis showed an association between systemic plasma levels of S100A8/A9 and outcomes, this relationship lost significance in multivariable logistic regression analysis when adjusted for established clinical predictors, including age, sex, NIHSS on admission, ASPECT Score on admission, and recanalization therapy (yes/no) (Ghasemi and Zahediasl, 2012). When using the NIHSS score 24 h post-admission instead of the baseline NIHSS, the odds ratio for S100A8/A9 further diminished, likely reflecting the robust predictive value of the 24-h NIHSS score.

Univariable and multivariable regression analyses of neutrophil counts and the neutrophil-to-lymphocyte ratio in relation to baseline characteristics yielded similar results regarding association and discrimination. This reinforces our hypothesis that S100A8/A9 is derived from neutrophils. Thus, in the context of predicting poor functional outcomes, the information provided by S100A8/A9 seems comparable to that offered by neutrophil counts or the neutrophil-to-lymphocyte ratio, both of which are easily accessible through routine differential blood counts.

Systemic plasma levels of S100A8/A9 at baseline have been reported to correlate with blood neutrophil counts (Cotoi et al., 2014). Our data are consistent with these findings, demonstrating a significant correlation between systemic plasma levels of S100A8/A9 and neutrophils, monocytes, and the neutrophil-to-lymphocyte ratio, although the strength of the correlation should be considered relatively low. Notably, the neutrophil-to-lymphocyte ratio has been reported as a prognostic biomarker (Bartt et al., 2022). In normal blood, neutrophils outnumber monocytes by more than tenfold and contain significantly higher levels of S100A8/A9, indicating that the majority of circulating S100A8/A9 originates from neutrophils (Edgeworth et al., 1991). Recently, elevated intravascular DAMP release has been identified in the hyperacute phase of stroke, correlating with infarct size and local leukocyte infiltration (Schuhmann et al., 2021b; Goyal et al., 2018). These findings support the role of S100A8/A9 in the inflammatory response following ischemic stroke and suggest its potential as a therapeutic target.

While our study demonstrates a strong association between elevated systemic plasma levels of S100A8/A9 and poor functional outcomes after ischemic stroke, it does not establish a causal relationship. The exact mechanisms by which S100A8/A9 may contribute to the development and progression of ischemic brain lesions are largely unknown. S100A8/A9 exerts its effector function and pro-inflammatory effects mostly via binding to toll-like receptor 4 (TLR4) and the receptor for advanced glycation end products (RAGE) (Vogl et al., 2007; Wang et al., 2018). RAGE is upregulated in endothelial cells upon hypoxia. Thus it is conceivable, but not proven, that soluble S100A8/A9 contributes to the blood-brain barrier disturbances during cerebral ischemia by stimulating local recruitment of additional leukocytes and induction of cytokine release (Wang et al., 2018). In myocardial infarction there is evidence that neutrophil-derived S100A8/A9 contributes to perpetuation of inflammation, myocardial injury but also resolution of inflammation (Sreejit et al., 2020). Moreover, S100A8/A9 can induce the formation of procoagulant platelets through GPIb-alpha and thereby foster fibrin formation and immune-driven thrombosis in diseases associated with high levels of S100A8/A9 e.g. stroke, as shown in our study (Colicchia et al., 2022). In the setting of sterile inflammation such as ischemic stroke and myocardial infarction the functional contribution of S100A8/A9 awaits further elucidation in appropriate animal models.

The main strength of our study is the prospective design, focusing on a cohort of 272 consecutive patients with moderate to severe AIS. This is an important aspect, because data from biomarker studies normally arise from patients with mild strokes (Montellano et al., 2021), although the prognostic role of biomarkers can differ between patients with mild and moderate to severe stroke (Montellano et al., 2024). Data collection was conducted in a blinded manner, ensuring objectivity in the predefined structured follow-up and biomarker assessment.

Our study has several limitations. First, although our sample size of 272 patients with severe ischemic stroke is robust for a focused study, it remains relatively small. To fortify the robustness and generalizability of our findings, future investigations should involve larger cohorts and replicate our findings externally. Validation through internal and external replication in independent datasets is essential for confirming our results. Second, systemic plasma levels of S100A8/A9 were measured at a single point in time after stroke. Biomarker trajectories created from repeated measurements may contain additional information regarding the development of functional outcome. Furthermore, blood samples were taken in median 27 [IQR: 20–37] hours after onset of symptoms. Third, pre-stroke systemic plasma levels of S100A8/A9 were unknown. Prior neurological conditions, e.g. unknown inflammatory conditions may have influenced the baseline levels, affecting our interpretations. Additionally, other potential confounders such as pre-stroke comorbidities, frailty, and baseline modified Rankin Scale (mRS) scores were not fully accounted for. These factors may influence both S100A8/A9 levels and functional outcomes. Moreover, S100A8/A9 might reflect systemic inflammation rather than directly mediating poor outcomes. Fourth, not all patients underwent mechanical thrombectomy and/or systemic thrombolytic therapy. Fifth, mRS at 3-month follow-up was assessed by telephone interviews and not during a personal visit. However, mRS assessment per telephone interview is a well-validated method of outcome assessment and the raters underwent previous certification (Baggio et al., 2014). Sixth, follow-up for patients with a poor outcome occurred a median of 5 days later than for those with a good outcome. However, despite this time difference, we believe there is no significant impact on the outcome.

## 5 Conclusion

In conclusion, we demonstrated that S100A8/A9 as a neutrophil-derived plasma biomarker was associated with functional outcome in patients with moderate to severe AIS. Its association with functional outcome after AIS as well as its correlation with neutrophil counts might underscore an important pathophysiological link between patients’ prognosis and stroke-related inflammation. However, these results need to be replicated in larger independent external cohorts before the role of S100A8/A9 as a central orchestrator of inflammation-associated morbidity in AIS can be fully judged.

## CRediT authorship contribution statement

**Christoph Vollmuth:** Writing – review & editing, Writing – original draft, Visualization, Validation, Supervision, Project administration, Methodology, Investigation, Funding acquisition, Formal analysis, Data curation, Conceptualization. **Felipe A. Montellano:** Writing – review & editing, Visualization, Validation, Methodology, Formal analysis, Data curation. **Cornelia Fiessler:** Writing – review & editing, Validation, Methodology, Formal analysis. **Fabian Essig:** Writing – review & editing, Visualization, Validation, Methodology, Formal analysis, Data curation. **Christian Hametner:** Writing – review & editing, Visualization, Validation, Methodology, Formal analysis. **Alexander M. Kollikowski:** Writing – review & editing, Visualization, Methodology, Formal analysis, Data curation. **Vivian Vogt:** Writing – review & editing, Methodology, Formal analysis, Data curation. **Mirko Pham:** Writing – review & editing, Visualization, Validation. **Peter U. Heuschmann:** Writing – review & editing, Validation, Project administration, Funding acquisition, Conceptualization. **Karl Georg Haeusler:** Writing – review & editing, Visualization, Validation, Investigation. **Guido Stoll:** Writing – review & editing, Visualization, Validation, Supervision, Funding acquisition. **Hermann Neugebauer:** Writing – review & editing, Validation, Project administration, Funding acquisition, Formal analysis, Data curation, Conceptualization. **Michael K. Schuhmann:** Writing – original draft, Visualization, Validation, Supervision.

## Ethics approval and consent to participate

Approval of the study protocol was obtained from the local Ethics Committee of the University of Würzburg, Germany (Reference No 05/20-am).

## Sources of funding

Christoph Vollmuth, Felipe A. Montellano, Fabian Essig and Alexander M. Kollikowski were supported by the German Research Foundation (Deutsche Forschungsgemeinschaft, DFG), Project No. 413657723 (Clinician Scientist-Programme UNION CVD), further Christoph Vollmuth was supported by the IZKF (interdisciplinary centre for clinical research), Z-3BC/11. Michael K. Schuhmann was supported by the Hentschel Stiftung. The Wuerzburger Stroke Cohort was funded by the Deutsche Forschungsgemeinschaft (DFG, German Research Foundation) – Project No. 374031971 – TRR 240.

## Declaration of competing interest

The authors declare the following financial interests/personal relationships which may be considered as potential competing interests: Karl Georg Haeusler reports speaker’s honoraria, consulting fees, lecture honoraria and/or study grants from Abbott, Alexion, Amarin, AstraZeneca, Bayer Healthcare, Biotronik, Boehringer Ingelheim, Boston Scientific, Bristol-Myers Squibb, Daiichi Sankyo, Edwards Lifesciences, Medronic, Novartis, Pfizer, Portola, Premier Research, Sanofi, SUN Pharma, and W.L. Gore and Associates. Peter U. Heuschmann reports grants from German Ministry of Research and Education, German Research Foundation, European Union, Federal Joint Committee (G-BA) within the Innovationfond, Charité–Universitätsmedizin Berlin, Berlin Chamber of Physicians, German Parkinson Society, University Hospital Würzburg, Robert Koch Institute, German Heart Foundation, University Göttingen (within FIND-AF [A Prospective, Randomised, Controlled Study to Determine the Detection of Atrial Fibrillation by Prolonged and Enhanced Holter Monitoring as Compared to Usual Care in Stroke Patients] randomized, supported by an unrestricted research grant to the University Göttingen from Boehringer-Ingelheim), University Hospital Heidelberg (within Registry of Acute Stroke Under Novel Oral Anticoagulants [RASUNOA]-prime, supported by an unrestricted research grant to the University Hospital Heidelberg from Bayer, Bristol-Myers Squibb, Boehringer-Ingelheim, Daiichi Sankyo), grants from Charité–Universitätsmedizin Berlin (within MonDAFIS, supported by an unrestricted research grant to the Charité from Bayer), outside the submitted work. Markus Otto served as scientific advisor for Axon, Biogen, Roche and Fujirebio. All other authors have nothing to declare. All other authors have nothing to declare.

## Data Availability

Data will be made available on request.
