# Supplementary material for: Systemic S100A8/A9 in patients with moderate to severe acute ischemic stroke: Exploratory analysis of inflammation and functional outcome
Source: Brain Behav Immun Health. 2025 Jun 18;47:101041. doi: 10.1016/j.bbih.2025.101041 (PMC12246696; doi:10.1016/j.bbih.2025.101041)
Supplement: Multimedia component 1 [file mmc1.docx]

**SUPPLEMENT MATERIAL**

**Systemic S100A8/A9 in patients with moderate to severe acute ischemic stroke: Exploratory analysis of inflammation and functional outcome**

Christoph Vollmuth^1^, Felipe A. Montellano^1,2^, Cornelia Fiessler^2^, Fabian Essig^1^, Christian Hametner^1^, Alexander M. Kollikowski^3^, Vivian Vogt^1^, Mirko Pham^3^, Peter U. Heuschmann^2,4,5^, Karl Georg Haeusler^6^, Guido Stoll^7^, Hermann Neugebauer^1^, Michael K. Schuhmann^1^

^1^ University Hospital Würzburg (UKW), Department of Neurology, Würzburg, Germany

^2^ University of Würzburg, Institute for Clinical Epidemiology and Biometry, Würzburg, Germany

^3^ University Hospital Würzburg (UKW), Department of Neuroradiology, Würzburg, Germany

^4^ Institute for medical Data Science, University Hospital Würzburg, Germany

^5^ Clinical Trial Centre, University Hospital Würzburg, Germany

^6^ University Hospital Ulm, Department of Neurology, Ulm, Germany

^7^ Institute of Experimental Biomedicine I, University Hospital Würzburg, Germany

**RESULTS**

**Baseline Characteristics and Differential Blood Count**

Patients with a poor functional outcome had significantly higher neutrophil counts [median: 7.3 × 10³/μl (IQR: 5.8–9.5)] compared to those with a good functional outcome [median: 6.4 × 10³/μl (IQR: 5.2–8.3), p=0.006] (**Figure S1A**). Similarly, patients with a poor functional outcome exhibited a significantly higher neutrophil-to-lymphocyte ratio (NLR) [median: 6.7 (IQR: 4.2–11.3)] compared to those with a good functional outcome [median: 5.0 (IQR: 3.3–7.1), p=0.006] (**Figure S1B**).

**Regression Analysis Results**

Table S2 summarizes the results of both unadjusted and adjusted regression analyses. In the unadjusted analysis: Higher neutrophil counts were significantly associated with poor functional outcome [odds ratio (OR): 1.16, 95% CI: 1.05–1.30] (**Table S2**). After adjusting for established predictors [age, NIHSS score 24 hours after admission, ASPECT score on admission, and recanalization therapy (yes/no)], this association was no longer significant [OR: 1.00, 95% CI: 0.86–1.17]. Higher NLR was also significantly associated with poor functional outcome in the unadjusted analysis [OR: 1.12, 95% CI: 1.05–1.20]. However, after adjustment for the same predictors, this association was no longer significant [OR: 1.02, 95% CI: 0.93–1.11].

**ROC Curve Analysis**

The ROC curve for predicting poor functional outcome (mRS 0–2 vs. 3–6) based on neutrophil counts had an area under the curve (AUC) of 0.62 (p=0.0015, 95% CI: 0.55–0.69) (**Figure S1C**). Similarly, the ROC curve for NLR predicting poor functional outcome showed an AUC of 0.63 (p=0.0006, 95% CI: 0.56–0.70) (**Figure S1D**).

**Subgroup analysis**

To assess pre-stroke frailty, we performed a subgroup analysis restricted to patients with a favorable pre-stroke functional status (mRS 0–2; see Table S3). Within this subgroup, patients with a poor outcome exhibited significantly elevated systemic plasma levels of S100A8/A9 [Median: 501 ng/ml (IQR: 340–908)] compared to those with a good outcome [Median: 397 ng/ml (IQR: 232–580); p = 0.008].

**Multicollinearity**

To assess potential multicollinearity, we examined the correlation matrix. Correlations greater than 0.8 or less than -0.8 may indicate multicollinearity, which was not the case in our analysis (table S4, Figure S2 and S3). Further we calculated the Variance Inflation Factor (VIF) values, as presented in Tables S4. All VIF values were below 5, indicating that multicollinearity is not a concern.

**Tables**

**Table S1:** Clinical, radiological, and biochemical data of the study population for patients with systemic S100A8/A9 levels > and < 565 ng/ml.

| **variable** | | | **biomarker level < 565 ng/ml** (n=162) | **biomarker level > 565 ng/ml** (n=110) | **p-value** |
| --- | --- | --- | --- | --- | --- |
| baseline data | | |  |  |  |
|  | age, years, median [IQR] | | 78 [65-84] | 80 [69-84] | 0.11 |
|  | sex, female, n (%) | | 77 (47.5) | 65 (59.1) | 0.06 |
|  | pre stroke mRS 0-2, n (%) | | 21 (13.0) | 28 (25.5) | **0.009** |
|  | creatinin, mg/dl, median [IQR] | | 0.94 [0.75-1.22] | 0.96 [0.82-1.27] | 0.17 |
| vascular risk factors, n (%) | | |  |  |  |
|  | hypertension | | 101 (62.3) | 74 (67.3) | 0.41 |
|  | diabetes mellitus | | 27 (16.7) | 27 (24.5) | 0.11 |
|  | heart failure | | 18 (11.1) | 17 (15.5) | 0.30 |
|  | atrial fibrillation | | 66 (40.7) | 56 (50.9) | 0.10 |
| stroke severity^a^, median [IQR] | | |  |  |  |
|  | NIHSS Score on admission | | 13 [8-17] | 13 [9-17] | 0.091 |
|  | NIHSS Score at 24h | | 8 [3-18] | 14 [7-23] | **0.0003** |
|  | NIHSS Score at 48h | | 6 [2-16] | 12 [5-21] | **0.0004** |
|  | NIHSS Score at 72h | | 4 [1-14] | 11 [4-17] | **0.0003** |
|  | NIHSS Score at discharge^b^ | | 2 [1-7] | 5 [2-11] | **0.001** |
|  | mRS at follow-up | | 3 [1-6] | 4 [3-6] | **<0.0001** |
| neuoradiologic data | | |  |  |  |
|  | blood vessel occluded, n (%) | |  |  |  |
|  |  | Carotis-T | 15 (9.3) | 13 (11.8) | 0.68 |
|  |  | M1 | 97 (59.9) | 67 (60.9) | 0.87 |
|  |  | M2 | 38 (23.5) | 20 (18.2) | 0.30 |
|  | ASPECT Score, median [IQR] | |  |  |  |
|  |  | ASPECT Score on admission | 8 [7-9] | 7 [6-8] | 0.068 |
| acute treatment, n (%) | | |  |  |  |
|  | thrombolysis | | 78 (48.1) | 40 (36.4) | 0.06 |
|  | time from onset to thrombolysis, hours^b^, Median [IQR] | | 1.6 [1.2-2.4] | 1.8 [1.3-2.4] | 0.84 |
|  | mechanical thrombectomy | | 129 (79.6) | 91 (82.7) | 0.53 |
|  | time from onset to mechanical thrombectomy, hours^b^, Median [IQR] | | 4.3 [2.4-8.8] | 4.2 [2.9-8.9] | 0.82 |
|  | TICI ≥ 2b | | 114 (70.4) | 79 (71.8) | 0.84 |
| blood sample and biomarker data | | |  |  |  |
|  | time from onset to blood sample, hours, Median [IQR] | | 27 [20-36] | 27 [20-39] | 0.64 |
| differential blood count, median [IQR] | | |  |  |  |
|  | thrombocytes × 10³/μl | | 188 [146-226] | 204 [156-253] | **0.042** |
|  | leukocytes × 10³/μl | | 8.8 [7.1-10.6] | 10.3 [8.5-12.6] | **<0.0001** |
|  | monocytes × 10³/μl | | 0.68 [0.54-0.86] | 0.79 [0.64-1.04] | **0.0005** |
|  | granulocytes | |  |  |  |
|  |  | eosinophiles × 10³/μl | 0.1 [0.0-0.1] | 0.0 [0.0-0.1] | **0.042** |
|  |  | neutrophiles × 10³/μl | 6.4 [5.2-8.4] | 7.9 [6.6-10.1] | **<0.0001** |
|  | lymphocytes × 10³/μl | | 1.3 [0.9-1.6] | 1.1 [0.7-1.5] | 0.07 |
|  | neutrophil-to-lymphocyte ratio | | 5.1 [3.5-7.8] | 7.4 [4.9-11.9] | **<0.0001** |
|  | a: deceased patients excluded | | | | |
|  | b: only patients with mechanical thrombectomy | | | | |

**Table S2:** Univariable and multivariable analysis for association of systemic plasma levels of S100A8/A9 and functional outcome 3-months after stroke.

| **variable** | **Odds ratio*** | **Odds ratio**** | **Odds ratio**** |
| --- | --- | --- | --- |
|  | **[95% CI]** | **[95% CI]** | **[95% CI]** |
| Age | 1.04 [1.02; 1.06] | 1.06 [1.03; 1.09] | 1.06 [1.02; 1.09] |
| NIHSS Score 24 h after admission | 1.25 [1.18; 1.34] | 1.22 [1.15; 1.32] | 1.22 [1.14; 1.31] |
| ASPECT Score on admission | 0.20 [0.11; 0.36] | 0.36 [0.16; 0.78] | 0.36 [0.16; 0.78] |
| Recanalization therapy (yes/no) | 0.61 [0.20; 1.57] | 0.60 [0.15; 2.19] | 0.58 [0.14; 2.15] |
| Neutrophil counts | 1.16 [1.05; 1.30] | 1.00 [0.86; 1.17] | / |
| Neutrophil-to-lymphocyte ratio | 1.12 [1.05; 1.20] | / | 1.02 [0.93; 1.11] |
| *Univariable analysis. | | | |
| **Multivariable analysis: adjusted for age, NIHSS 24 hours after admission, ASPECT Score on admission and recanalization therapy (yes/no). | | | |

**Table S3: Subgroup analysis of patients with good pre-stroke functional status (mRS 0–2).**

| **variable** | | **mRS 0-2**  **at 3 months** (n=80) | **mRS 3-6**  **at 3 months** (n=142) | **p-value** |
| --- | --- | --- | --- | --- |
| biomarker data, median [IQR] | |  |  |  |
|  | S100A8/A9, ng/ml | 397 [232-580] | 501 [340-908] | **0.008** |

**Table S4:** Assessment of multicollinearity among variables included in the regression analysis.

| ***variable*** | ***VIF*** | ***R2 with other variables*** | ***VIF*** | ***R2 with other variables*** |
| --- | --- | --- | --- | --- |
| *Age* | *1.25* | *0.20* | *1.25* | *0.20* |
| *Sex* | *1.16* | *0.14* | *1.15* | *0.13* |
| *NIHSS Score on admission* | *1.17* | *0.15* | */* | */* |
| *NIHSS Score after 24h* | */* | */* | *1.25* | *0.20* |
| *ASPECT Score on admission* | *1.14* | *0.13* | *1.21* | *0.17* |
| *Recanalization therapy (yes/no)* | *1.04* | *0.04* | *1.04* | *0.04* |
| *log(S100A8/A9)* | *1.02* | *0.02* | *1.02* | *0.02* |

**Figures**

**Figure S1:** Prognostic value of neutrophil counts and the neutrophil-to-lymphocyte ratio in terms of stroke severity to predict 3-months outcome. **A)** Neutrophil counts according to 3-month functional outcome (good vs. poor outcome). **B)** Neutrophil-to-lymphocyte ratio according to 3-month functional outcome (good vs. poor outcome). **C)** ROC analysis for 3-months functional outcome (mRS 0-2 vs. mRS 3-6). **D)** ROC analysis for 3-months functional outcome (mRS 0-2 vs. mRS 3-6).


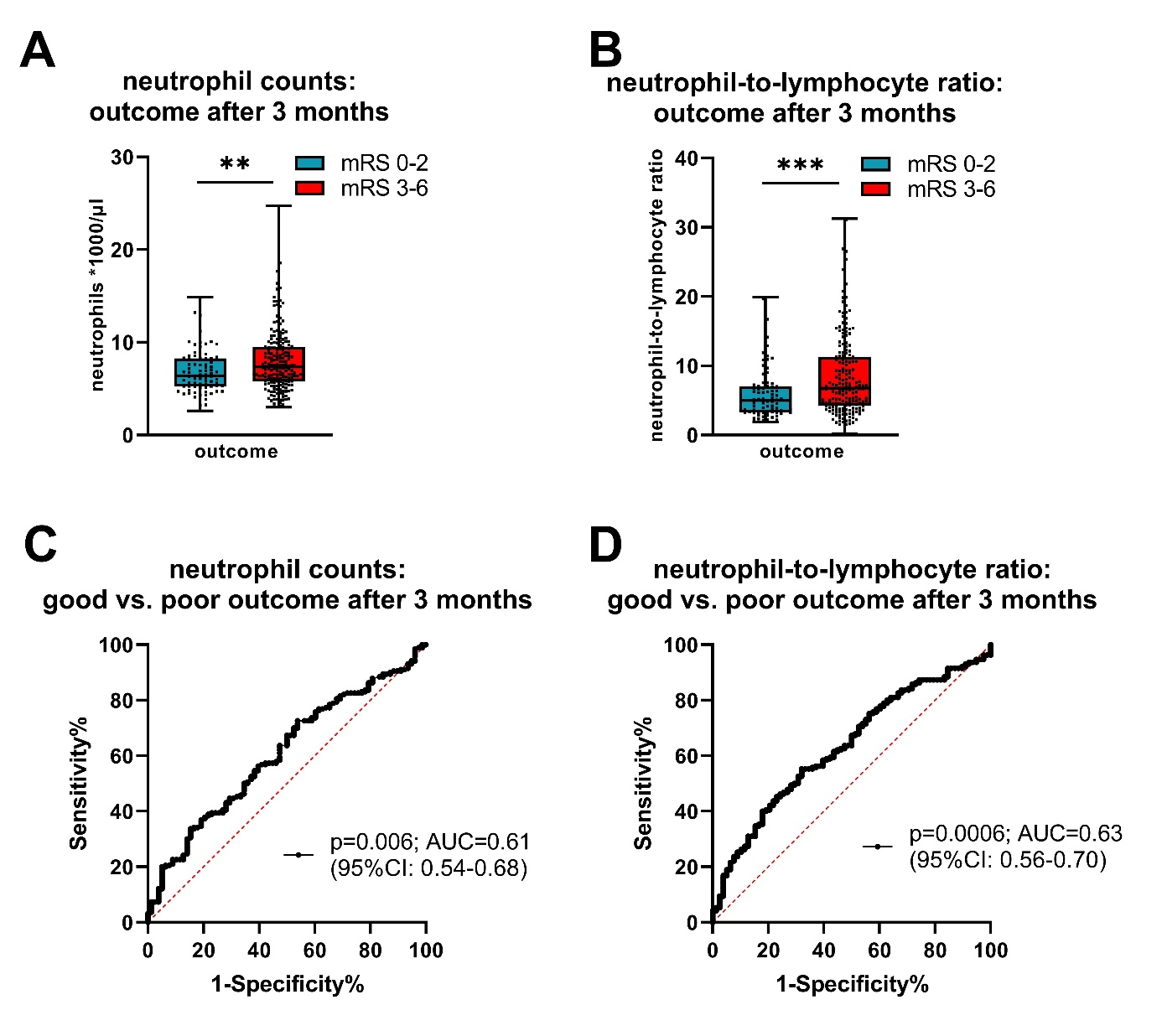


***
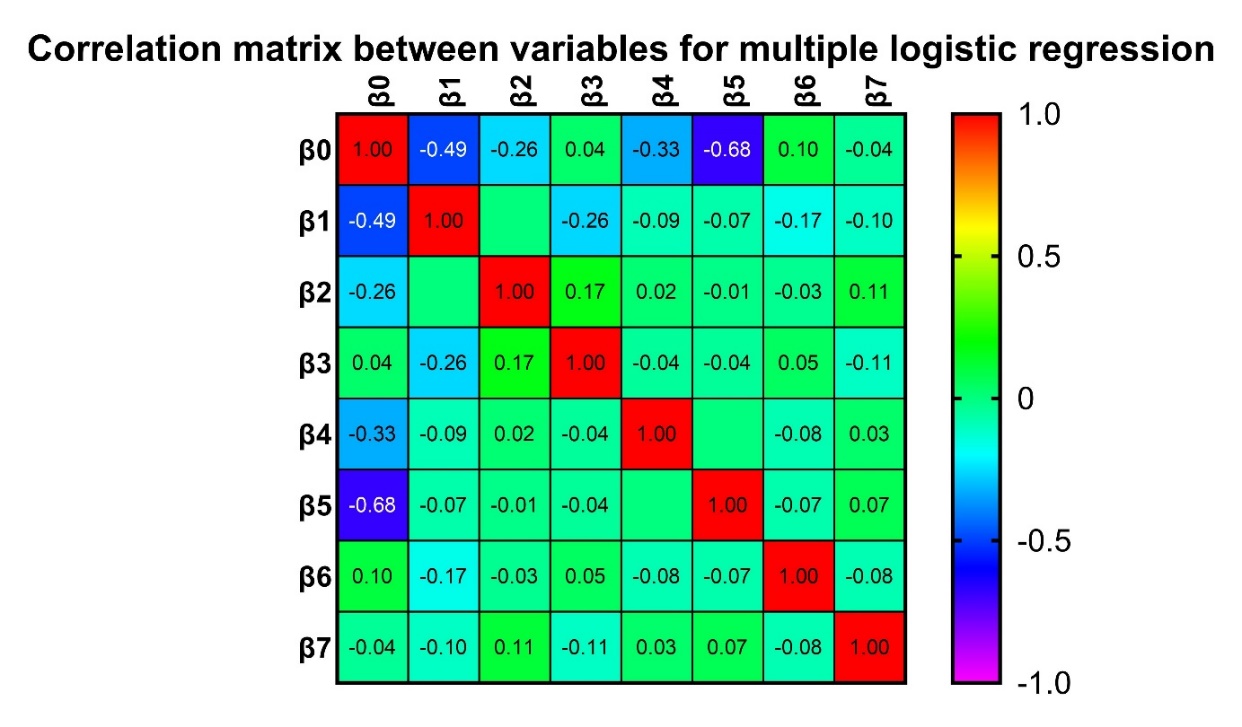
*Figure S2:** Correlation matrix between the following variables for multiple logistic regression: β0 Intercept, β1 age, β2 NIHSS Score on admission, β3 ASPECT Score on admission, β4 recanalization therapy (yes/no), β5 log(S100A8/A9), β6 sex, β7 mRS pre stroke.

***
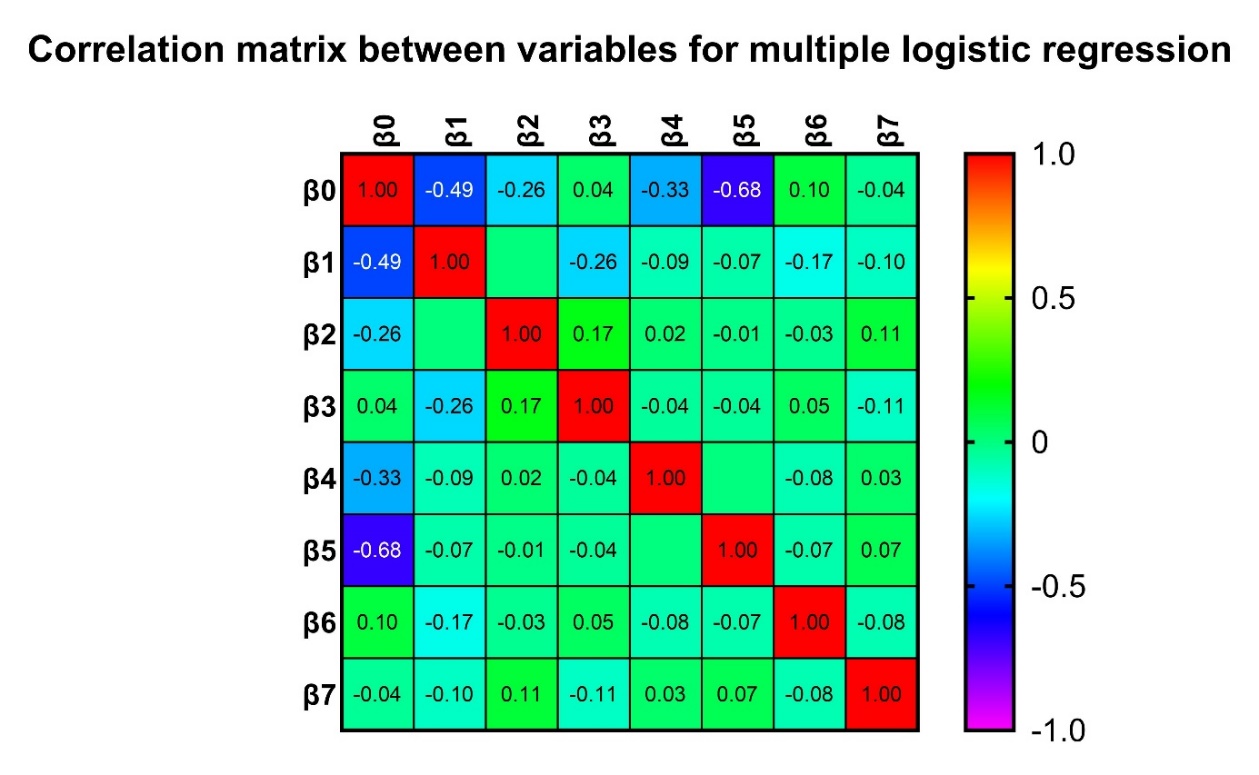
*Figure S3**: Correlation matrix between the following variables for multiple logistic regression: β0 Intercept, β1 age, β2 NIHSS Score 24h after admission, β3 ASPECT Score on admission, β4 recanalization therapy (yes/no), β5 log(S100A8/A9), β6 sex, β7 mRS pre stroke.
